# Biobased polyurethanes for biomedical applications

**DOI:** 10.1016/j.bioactmat.2020.10.002

**Published:** 2020-10-15

**Authors:** Sophie Wendels, Luc Avérous

**Affiliations:** BioTeam/ICPEES-ECPM, UMR CNRS 7515, Université de Strasbourg, 25 Rue Becquerel, 67087, Strasbourg Cedex 2, France

**Keywords:** Polyurethanes, Biobased, Biomedical, Bioactive, Tissue engineering, Biocompatibility, Scaffold

## Abstract

Polyurethanes (PUs) are a major family of polymers displaying a wide spectrum of physico-chemical, mechanical and structural properties for a large range of fields. They have shown suitable for biomedical applications and are used in this domain since decades. The current variety of biomass available has extended the diversity of starting materials for the elaboration of new biobased macromolecular architectures, allowing the development of biobased PUs with advanced properties such as controlled biotic and abiotic degradation. In this frame, new tunable biomedical devices have been successfully designed. PU structures with precise tissue biomimicking can be obtained and are adequate for adhesion, proliferation and differentiation of many cell's types. Moreover, new smart shape-memory PUs with adjustable shape-recovery properties have demonstrated promising results for biomedical applications such as wound healing. The fossil-based starting materials substitution for biomedical implants is slowly improving, nonetheless better renewable contents need to be achieved for most PUs to obtain biobased certifications. After a presentation of some PU generalities and an understanding of a biomaterial structure-biocompatibility relationship, recent developments of biobased PUs for non-implantable devices as well as short- and long-term implants are described in detail in this review and compared to more conventional PU structures.

## Highlights

• Detailed last trends in biobased, biocompatible and biodegradable PUs.
• A comprehensive study of “structure-biocompatibility relationships” on PU.
• A rigorous analysis of biobased PUs as wound dressing materials.
• The performances for short- and long-term implants were carefully analyzed.
• An insight into the shape-memory materials for biomedical applications.

## Graphical abstract

**Figure:**
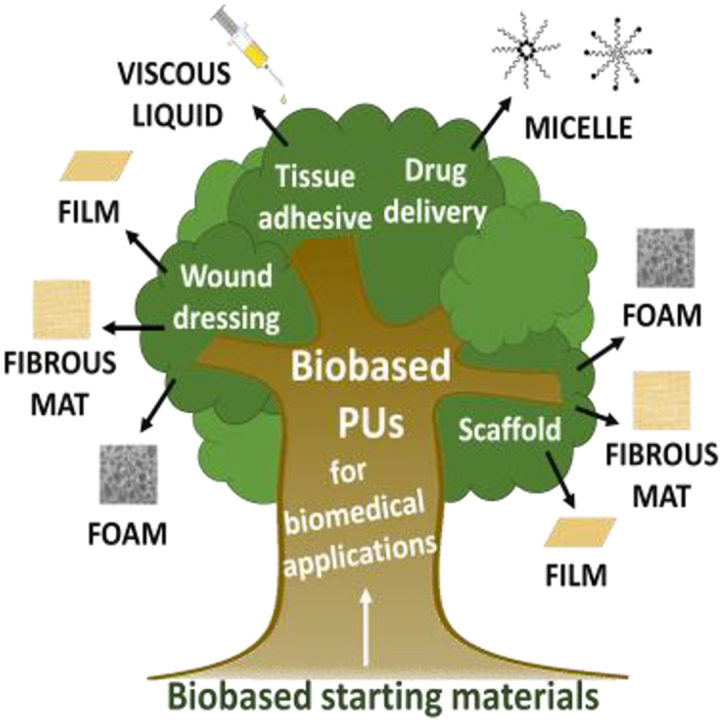

## 1 Introduction

Today, engineered smart biomaterials are designed to mimick tissue properties and behaviors and are displaying enhanced properties such as cell adhesion and proliferation. In this way, Different polymeric materials can be used, either as coating or as plain medical devices such as polyvinylidene fluoride (PVDF), polyethylene (PE), polypropylene (PP), poly (methyl methacrylate) (PMMA) and silicone, only to cite a few of them [1]. In the last decades, more and more biocompatible PUs have also found applications in the biomedical area thanks to their tunable properties and behaviors. Medical grades PU-based polymers are found under several tradenames such as Carbothane™, Pellethane® or Tecoflex™ from Lubrizol (US), or Carbosil® and Bionate® from DSM (Holland). These different fossil-based PUs reflect this rich and prosperous market since they have gained their trusts among physician's and surgeon's communities. Mostly thermoplastic-based, sometimes blended with other polymers, they can be shaped and modified according to the targeted application [[2], [3], [4]]. More and more, materials answer to the growing need for new, durable or sustainable solutions which are linked to several factors such as a global worldwide trend towards biobased and green solutions [5]. To answer to these strong needs and trends, competitive properties have been mainly achieved through different new biobased macromolecular architectures. However and till now, renewable PU are yet still a niche market with around 0.1% in the approximately 20 million tons of PU produced per year [6], taking into account that among these 20 million tons, less than 3% correspond to the PU production for biomedical devices and applications [7]. To be accepted and validated for biomedical applications, PUs should, as all types of materials in this field, follow a long process and fulfill some strict requirements in terms of cytotoxicity, acute and subchronic toxicity, as well as hemocompatibility and carcinogenicity. These requests depend on the i) targeted living tissue in contact and ii) the estimated time of contact with the living tissues. These requirements are described in different norms, such as the ISO 10993 for biological evaluation of medical devices which is nowadays the international standard for biocompatibility evaluation, approved by the U.S. Food and Drug Administration (FDA) and the European CE label [8].

This review aims at reporting the recent progress on biobased PUs preparation for non-implantable devices, as well as short- and long-term implants applications. For that, generalities comprising some definitions, an overview of the PU chemistry, synthetic pathways and safety concerns were first introduced. Then, a detailed examination of the structure-biocompatibility relationship and the corresponding improvement methods was conducted, followed by a description of PUs general biomedical applications as non-implantable, short- and long-term devices. Last innovations on biobased PUs structures and properties for these applications were subsequently analyzed in detail. Finally, attention will be drawn to the remaining challenges and future perspectives on the subject as conclusion.

## 2 Generalities

### 2.1 Some definitions

Precise definitions in this field are crucial. In connection with IUPAC definitions [9], some basic definitions are given, below.

A biomaterial can be described as “any substance or combination of substances, other than drugs, synthetic or natural in origin, which can be used for any period of time and augments or replaces partially or totally any tissue, organ or function of the body”, by the American National Institute of Health [10].

Biobased polymer/material, also described as biomass/renewably sourced, refers to the origin of the carbon and main atoms. It is a material from which the starting components are, directly or not, derived from the biomass (systems produced by living organisms: vegetal, animal, fungi). In this regard, a biomacromolecule is a macromolecule which is directly extracted from biomass. Biopolymers are based on biomacromolecules, as for example Polyhydroxyalkanoates (PHAs). To evaluate the biobased content of biobased materials, several certifications around the world exist. These labels are based on the measurement of biobased content, usually by ASTM D6866, ISO 16,620-2 or EN 16640/EN 16785. They consist for instance on the radiocarbon analysis for the determination of the biogenic 14C content [11]. Some examples of labels and their required minima biobased contents are listed in Table 1. In this overview as in a concern of simplicity, a PU is considered biobased when the whole material is at least 20 wt% biobased or potentially biobased.

**Table 1:** Some labels and the corresponding minimum biobased content [11].

Region	Label name	Norm	Minimum biobased content required
Austria	Vinçotte OK Biobased	EN 16785-1	20%
Germany	DIN CERTO DIN-Geprüft Biobased	ASTM D6866	20%
Korea	Korea Bio Material Packaging Association's Biobased Label	ASTM D6866	25%
USA	Roundtable on Sustainable Biomaterials (RSB)	ASTM D6866, EN 16,640, ISO 16620	25%
European union	EU Ecolabel	EN 16087, EN 16640, EN 16785-1	25%

A biodegradable polymer/material first refers to its ability to be decomposed in the environment mainly by enzymatic action of microorganisms, in aerobic or anaerobic conditions [12,13]. For polymers and biopolymers, this biotic degradation mostly results for instance in chain cleavage with CO2 and CH4 production, alongside the production of water and a new biomass under precise standard conditions (for instance EN 13432). By extension, a biodegradable (or bioresorbable) polymer/material for biomedical application refers to its ability to be largely decomposed by a biotic mechanism in contact with living tissue, in contrary to an abiotic degradation.

The term “biocompatibility” should be understood by the capacity of the tissues to be brought in direct contact with a material without causing a systemic toxic response or other adverse effect on biological system [14] such as an allergic response, inflammation or infection for example. Biocompatibility description regarding cell-biomaterial interactions is described in part 3 of this work. In this frame, a bioactive material refers to a biocompatible material capable of provoking a (beneficial) surrounding tissue(s) response. As the opposite of a bioactive material, a bioinert material is biocompatible but does not induce a response from its surrounding environment.

**Fig. 1:**
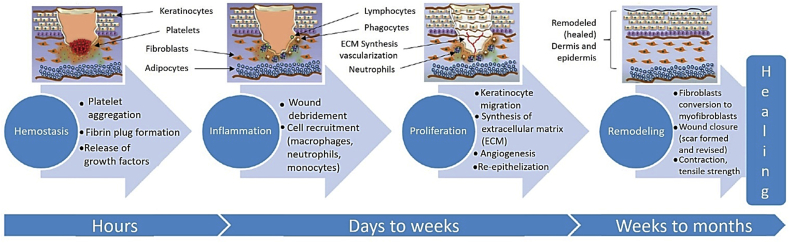
The four main stages of wound healing, adapted from Ref. [15].

According to the U.S. Food and Drug Administration (FDA), implants are devices that are placed inside the body or on the body surface. They can be placed permanently or not. In this work, long-term implants refer to implants that have a 30 days or more lifetime in contact with the tissues before biodegradation or removal, to assist healing until the tissue remodeling phase, as described in Fig. 1. In contrast, short-term implants have less than 30 days lifetime in contact with living tissues. Finally, non-implantable devices are materials designed to be placed on the body surface.

### 2.2 Polyurethane chemistry

#### 2.2.1 Historical outline

The first PU described in the literature was related to Otto Bayer's works in 1937 on the polyaddition reaction between a diisocyanate and a polyester diol. This discovery allowed PU uses during World War II, especially as adhesives, foams and fibers for military equipment and weapons [16]. At the end of the war, PUs started to be produced at industrial level for other types of applications, mainly as adhesives, elastomers or coatings. Commercial productions of PU foams, especially flexible foams, only started in the 1950's. At the same time, PU properties were assessed for various types of medical applications, including bone fixation [17], coatings [18] and artificial heart [19]. Since then, the success and versatility of PU properties for biomedical applications generated an extensive use of this polymer in many biomedical fields [[20], [21], [22], [23]]. However, behind the apparent biocompatibility of PU-based materials and since the beginning of their uses as biomaterials, the isocyanate toxicity, particularly in the case of aromatic isocyanates, has been considered as a major drawback. The question of aromatics uses in PU formulations and their corresponding impacts are analyzed in detail in this review, below in 2.2.4.

Nowadays, the two principal diisocyanates used at industrial scale are oligomeric 4,4′-Methylene diphenyl diisocyanate (MDI) and Toluene diisocyanate (TDI) because of their cost-efficiency as well as higher reactivity, mechanical resistance, biostability and abrasion resistance, for example. Due to these qualities, PUs production types are nowadays numerous and the main ones include rigid and flexible foams, coatings, adhesives and sealants and elastomers [7] for a very large panel of applications from insulation to bedding, paints, in the automotive and footwear industry or, in the frame of this study, for biomedical purposes. During the last decades, the development of chemicals derived from vegetable oils and starch has raised interest for the elaboration of biobased polymers, including for PUs. This interest goes hand in hand with a raising global environmental concern on plastic wastes and a need to create more sustainable materials. To answer to this concern, the PU industry has since then been focusing on replacing PUs, step by step, starting materials with biobased equivalents, or replaced with new biobased macromolecular architectures. Improving the process for PU preparation in a greener way has also emerged as a strong sustainable solution to answer to current concerns.

#### 2.2.2 Synthesis and chemical structures

**Fig. 2:**
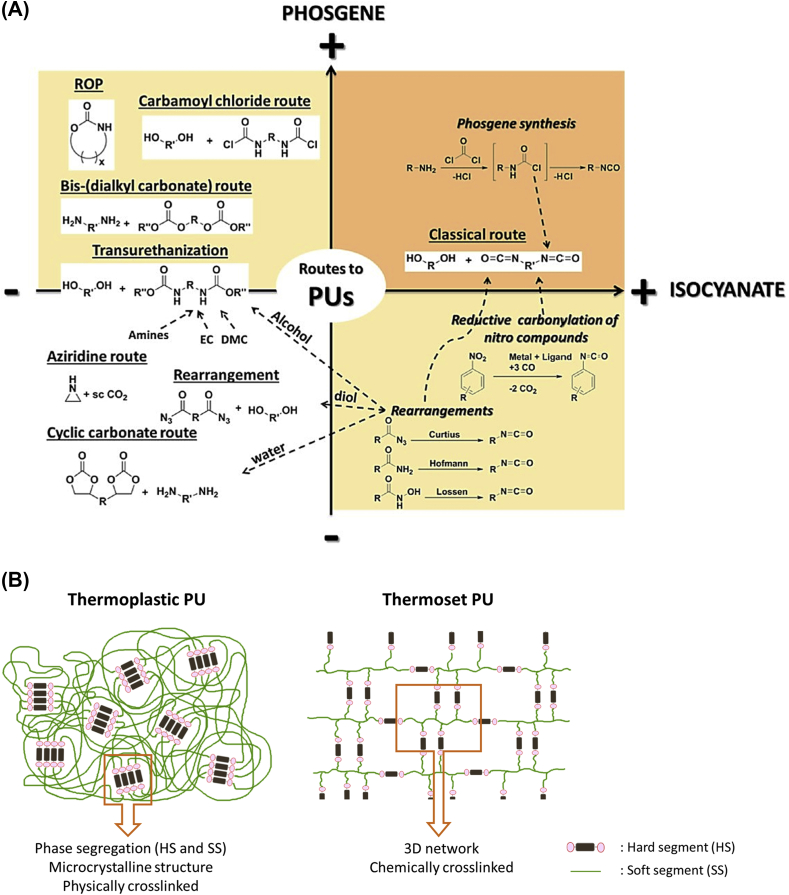
(A) Overview of the main synthetic routes to PUs [24] and (B) schematic representation of a TPU and a thermoset PU.

Up until now, many synthetic routes to PUs have been described and investigated, as depicted in Fig. 2 [24]. The main and major way is the reaction between a polyol and a polyisocyanate via a polyaddition reaction. The key point with this path is the high reactivity of the isocyanate group –NCO against labile hydrogen from active compounds such as hydroxyl, water, carboxylic acids and anhydrides, amines and thiols, as well as urethane and urea [25]. Alternatives to the use of toxic isocyanate monomers have also been explored, more particularly during the last decade. The obtained materials from isocyanate-free routes are called non-isocyanate polyurethanes (NIPUs). Several studies have shown the potential of NIPUs as alternative to the classic PU synthetic route, with numerous articles and reviews [24,[26], [27], [28]]. Very briefly and nowadays, NIPUs can be mainly obtained with three synthetic pathways: transurethanisation, aziridine copolymerization and cyclocarbonate aminolysis. This latter one is the most used and studied. However, NIPUs presents several drawbacks such as (i) a slow kinetic (sometimes several days are needed for the complete synthesis) and (ii) PUs with low molar masses, compared to isocyanate-based PUs, are obtained.

All PU structures can be divided into two principal families which are (i) thermosets and (ii) thermoplastic polyurethanes (TPUs). TPUs consist of linear and uncrosslinked structures synthesized from bifunctional alcohols (diols) and diisocyanates. They are mostly obtained by a two-steps process. The first step is based on the synthesis of a prepolymer with –NCO ending chains by reaction of a long diol such as a polyester or a polyether-diol, with an excess of diisocyanate (at least 2:1 NCO:OH molar ratio). In a second step, long TPU chains are obtained using a chain extender (typically a short diol as the 1,4 Butanediol (BDO)) and the previous prepolymer. This procedure allows a fine control on the final linear structure. TPUs can also be prepared in a one-step method by mixing all the components (diol, diisocyanate and chain extender), this time without control on the final macromolecular architecture. TPUs are soluble and thermo-reprocessable materials.

In opposite, thermoset PUs are mainly prepared by mixing polyols and polyisocyanates in a one pot process such as for PU foams. In this case, at least one of the monomers has a functionality of 3 or more, which leads to chemically crosslinked 3D-networks. However, thermoset PUs can be prepared in some cases by a two-step method. Common foams formulations with or without open cells for rigid to soft foam, respectively, require at least five reagents such as a polyisocyanate, a polyol, a catalyst system, a surfactant and a blowing agent. In opposition to TPUs, the chemically crosslinked organization make it unable for these materials to be thermo-reprocessed or soluble. In answer to this issue, new reversible crosslinked networks have been elaborated based on dynamic covalent bonds that can disconnect and reconnect with or without the use of a stimulus [29]. They are called covalent adaptable networks (CANs) and are classified in two categories according to their exchange mechanism: dissociative and associative networks [30]. Associative networks such as for example vitrimers are processable without depolymerization and with limited properties loss [31,32].

In PUs, and even more specifically in TPUs, several phases from micro-segregations can be observed between the so-called hard segment (HS), usually based on rigid diisocyanate and the chain extender, and the soft segment (SS) which is mainly based on a flexible and long diol. This segregation into organized domains is due to physical interactions between PU chains in the form of hydrogen-bonding between urethane functions. Schematic representation of the TPU microcrystalline structures and of a chemically crosslinked thermoset PU is depicted in Fig. 2. Final PUs properties can be then tailored according to the polyol nature, architectures and molar masses, as well as the diisocyanates involved in the synthesis.

In order to achieve high conversion rates and high molar masses for isocyanate-based PU or NIPU synthesis, the use of catalysts is often required. Among them, organometallic catalysts (especially organotin) and/or tertiary amines [33] seem to be the most effective ones, but organo-catalysis using organic acids and bases has also been explored in the last years [34]. However, we can notice that it is challenging to remove organotin-based catalysts from the matrix. They are often kept in the polymer, which may cause significant tissue response and toxicity in the case of PU elaboration for biomedical applications [35,36].

#### 2.2.3 Renewable PUs from diverse biomasses

In the last two decades, the research has been focused on the development of new biobased macromolecular architectures with competitive properties compared to fossil-based polymers. For that, new starting materials (biobased building blocks) have been developed for PU synthesis from different bioresources. The main currently available sources of biomasses are polysaccharides (starch, cellulosic derivatives, chitin, alginate), animal and vegetal proteins, lipids and molecules produced by microorganisms obtained by white biotechnology from bioproduction. Existing or new biobased molecules can be obtained. Then, new buildings blocks can be used for the synthesis of PU from a large range of new architectures such as biobased polyesters [37] from acids and polyacids, aliphatic and aromatic [38,39] polyols [25,37], amines [40], epoxy [41] or furans [42]. Moreover, the development of mono- and polyisocyanates derived from biomass has recently gained substantial interest for the synthesis of fully biobased PUs [25,[43], [44], [45]]. In the case of NIPU synthesis, different new biobased cyclocarbonates [26] and renewable polyamines can be used.

However, the biobased content is just a rule of the well-known twelve principles of green chemistry [46]. The extraction of biomass and their modifications must allow the use of green solvents and catalysts, low energy consumption without a loss in atoms economy. To promote more sustainable synthetic ways, the elaboration of greener catalysts [47] and solvents [48] have then become major research topics. However, their uses for the synthesis of PU is still now limited. However, efforts are performed in PU syntheses without solvents and catalysts [49]. Renewable polymers offer diverse new materials for uses in biomedical fields. Biobased polymers and biopolymers such as polysaccharides [50], polylactic acid (PLA) [51] or PHA [52,53] have shown great biocompatibility, cytocompatibility and degradation properties compared to some fossil-based polymers and have proven suitable candidates for biomedical application. However, the lack of adaptable mechanical properties, and sometimes excessive swelling hinders their uses for specific applications. Therefore, their incorporation into PU backbones seems to offer advantages in terms of improved and tunable properties. Their modifications and further incorporations in PUs are discussed below in a dedicated part.

#### 2.2.4 PUs safety and biocompatibility concerns

**Fig. 3:**
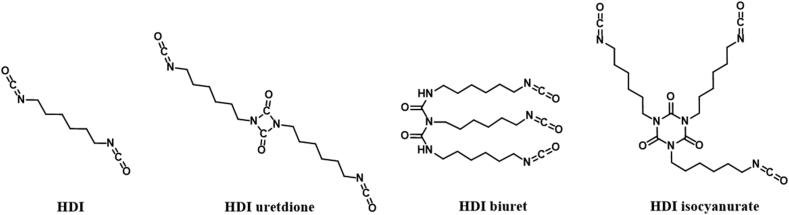
HDI and oligomeric HDI chemical structures, from Ref. [57].

Despite the generally accepted PU biocompatibility, several safety concerns regarding each step of PU synthesis remain such as (i) the toxic polyisocyanate synthetic pathway using for instance phosgene, a highly toxic gas at room temperature [54], (ii) the impurities in the prepared polyols and polyisocyanates, and (iii) the residual unreacted polyisocyanate monomer, catalysts and/or additives in the final material. Indeed, one of the major concerns linked to unreacted polyisocyanates is their own mutagenic and toxic behavior [55], especially for highly reactive MDI and TDI with their conjugated benzene rings. Main polyisocyanates exposure routes are through respiratory and dermal contact while for instance manipulation for PU synthesis [56]. Besides, polyisocyanates are irritant and sensitizers even at low concentration, due to the high –NCO reactivity and exothermic reaction with soft tissue, water and amino acids, giving in a first step toxic polyamine. Besides, due to monomers with low molar masses and high vapor pressures (especially for TDI and HDI [56]), many cases of occupational asthma have been reported due to contact with polyisocyanates [57,58]. However, despite many techniques developed for isocyanate dermal exposure detection, reliable quantitative results are still challenging to be obtained due to their high reactivity, in contact with the skin [59]. Thus, purity of the different starting materials, a well-controlled NCO:OH ratio and additives contents play critical roles in PU biocompatibility. Other methods lowering the polyisocyanate monomer reactivity comprise lowering their volatilities by the use of oligomeric derivatives with higher molar masses, as depicted in Fig. 3 for HDI [57,60]. These oligomers are prepared from isocyanate monomers reaction with one (uretdione) or two (isocyanurate) other isocyanate monomer equivalent, or with a disubstituted urea (biuret) [25]. Non-aromatic alternatives are also appreciated, such as MDI's aliphatic derivate the 4,4′-methylenebis (cyclohexyl isocyanate) (HMDI) or isophorone diisocyanate (IPDI). An elegant approach allows to avoid the use of polyisocyanates with NIPU architectures elaboration. However and until now, for medical-grade, MDI-based PUs and its oligomeric counterparts are still the most common used polyisocyanates.

Regarding PU usages as biomaterial, degradation in contact with living tissues remains another concern due to the engendered loss of device performance and longevity, as well as harmful molecules release. In fact, given a certain time at body temperature and tissue contact, PU will undergo hydrolytic [[61], [62], [63]] (catalyzed or not by acidic or basic pH), enzymatic [62,64], oxidative [61] (as a result of cell biological response to implantation of foreign materials) and physical degradation [61] (mainly due to water absorption) that further lead to surface erosion, cracks and ultimately to biomaterial failure. Main chemical functions found in PU chains and their sensitivity to different types of biotic or abiotic degradations are listed in Table 2. Degradation results in the release of various types of molecules that can alter tissue environment and cause for instance local inflammatory response. SS degradation mainly leads to the formations of small-chains hydroxyls, carboxylic acids, aldehydes or ketones [65], while biotic HS degradation by enzymatic action produces polyamines [64] that can further undergo oxidative degradation or be moved to liver and kidney for elimination [66]. Thus, a well control of degradability by varying the chemical architectures linked to the SS and HS parts, is crucial to maintain PU biocompatibility over time and to avoid tissues sur-exposition to toxic and carcinogenic diamines such as for instance MDA and TDA linked to HS degradation.

**Table 2:** Main PU functions and their principal degradation types.

Functions/Polymer	Main degradation types
Ester/Polyester	Hydrolytic [61], enzymatic [62,64]
Ether/Polyether	Oxidative [61], enzymatic, physical [61]
Carbonate/Polycarbonate	Enzymatic [62,64], hydrolytic [61] a
Polyamine	Oxidative [61]
Urethane HS/Polyurethane	Enzymatic [62,64], hydrolytic [61] a
Urea HS/Polyurea or Polyurethane-urea	Enzymatic [62,64]

a Lower than ester functions but higher than ether.

This review is mainly divided in three main sections. First, focus was placed on understanding general biomaterial- and PU structure-biocompatibility relationships with specificity on cell adhesion mechanisms on biomaterials, followed by the presentation of the different approaches for increasing PU bioactivity. Then, conventional PUs (fossil-based) were investigated for biomedical applications. Finally, the elaboration of renewable PUs for non-implantable medical devices as well as short- and long-term implants were developed and analyzed in detail.

## 3 General approaches and analysis of the structure-biocompatibility relationships

To understand better conventional or biobased PU biocompatibility, one needs to acknowledge that the biomaterials interaction with the living tissue is a key parameter which induces modulable integration. This keypoint is linked to induced cell adhesion, proliferation and migration on the material surface at the interface. A lower interface quality can indeed be responsible for local infections and different drawbacks such as inflammations. In their environment, cells adhere to components of the extracellular matrix (ECM) like collagen via cell receptor molecules known as integrins and located on their surface. The ECM is a proteins-based three-dimensional network, from which the most important for cell adhesion and migration being collagen and glycoproteins such as fibronectin that possess cell-binding sites [67]. Thus, cells mainly adhere to the ECM through chemical and biological connections [68], but also through ECM physico-chemical properties at the interface such as surface roughness, wettability and free energy [69]. Cell-ECM adhesion also comprise physical interactions at the interface, which are classified by distance. The so-called long-range interactions involve van der Waals forces and electric repulsions, while short-range forces comprise hydrogen bonds, dipole interactions and hydrophobic interactions [68,70]. In addition to that, ECM composition, architecture and properties adapt to its location, hence each tissue type has its own ECM [69]. It also transmits intracellular signals able to trigger cell proliferation, migration and differentiation and is considered the biological support for growing cells. An ideal biomaterial intended for tissue reconstruction should then mimic tissue specific ECM architecture, with adequate chemical and physico-chemical properties at the surface [71], to favorably interact at the interface with surrounding cells to further trigger their adhesion, proliferation, migration and, when needed, differentiation. On the contrary, for some applications requiring direct blood contact and to avoid thrombosis and embolism, biomaterials surface should be developed to avoid cell and protein (for example fibrinogens and albumin) adhesion and then clot formation [68]. Therefore, engineering PU-based materials mean adapting its architecture, and its chemical and physico-chemical properties for example, hydrophobic/hydrophilic balance, HS and SS structures and contents, water adsorption, as well as surface free energy and thickness [72,73]. For example, according the systems, endothelial cells have demonstrated opposite behaviors with two different PU types [74]: they showed high proliferation in a continuous single layer on a polyester-based PU [74], or in a limited area for polyether-based PU [75].

Improvement of mesophilic bacterial adhesion resistance at the tissue-material interface has been a challenge since the early stages of biomaterials with many cases of infections with medical devices and implants, especially due to *Staphylococcus aureus* (*S. aureus*). In fact, bacteria cells are capable to adhere and proliferate on a surface via the same mechanisms than human body cells but in a shorter amount of time, sometimes only a few minutes are enough to cause a wound infection [76]. Furthermore, they are capable of PU degradation [64]. In most cases, they adhere preferably on PU via its hydrophobic segments with the corresponding chemical groups. To avoid mesophilic bacterial and protein adhesion onto PU surfaces, as well as confer PU architecture other bioactive properties, many approaches have been developed to insert bioactive compounds into the PU, as described below.

Bioactive PUs have been elaborated either (i) to be directly used as implants or non-implantable biomaterials, or (ii) to be used as coatings to improve the biocompatibility at interface of implants/non-implantable biomaterials based on PUs or other polymers, metals, or ceramic, for instance.

### 3.1 Active compound in the backbone

This section is focused on the incorporation of active compounds directly into the PU backbone. With this approach, not only PUs access intrinsic antibacterial, anti-inflammatory [77] and/or antiplatelet adhesion properties [78], but in the case of implants, the bioactive compound can also be released during PU biodegradation, allowing a stable compound release over time [79]. In order to increase PU antimicrobial activity, known bactericide with initial active hydrogen functionalities might be used, such as chloramphenicol [80] or metal derivatives such as cobalt (II) hydroxide Co(OH)2 [81]. Quaternary ammonium salts (QAS) are also proven to be bacterial adhesive inhibitors. Many chemical groups containing QAS with mono- or bi-functional end-groups such as hydroxyls [[82], [83], [84]] or amines [85,86] can be used to be further incorporated into the HS of PUs as chain extender or chain terminer [[87], [88], [89]]. Besides QAS biological activity, their antimicrobial efficiency mainly depends on the number of nitrogen-based ions, the length and nature of its tail and the corresponding counter ion. Of course, increasing the number of ammonium generates higher antibacterial properties, as well as extending the chain length of alkyl [82] or PEG-based [83] chains tails. However, slightly better cytocompatibility was observed for shorter alkyl chain length [85]. Prepared bactericide QAS-diol combined with zwitterionic functionality exhibited antimicrobial and non-hemolytic activity [88] or non-specific protein adsorption resistance [90]. Recently, so-called Gemini QAS (GQAS), or dimeric QAS (containing two nitrogen ions), were inserted into waterborne PU (WPU) backbone and exhibited enhanced antibacterial properties due to the presence of multiple nitrogen-based ions [91]. Their chemical structures are presented in Table 3. Besides, WPU with biobased lysine-derivative GQAS containing hydrophilic heads and different hydrophobic alkyl chain lengths allowed the formation of antibacterial layers on the material surface and tunable degradability [85].

**Table 3:** Main chemical structures of QAS and their properties.

Ref	QAS Structure	Properties	Bacteria tested
[82]		Antimicrobial	*E. coli*
			*S. aureus*
			B. subtilis
[83]		Antimicrobial	*E. coli*
			*S. aureus*
[84]		Antimicrobial	*E. coli*
			*S. aureus*
[85]		Gemini-QAS	*E. coli*
		Antimicrobial	*S. aureus*
[86]		Gemini-QAS	*E. coli*
		Antimicrobial	*S. aureus*
[87]		Antimicrobial	*E. coli*
			*S. aureus*
			B. subtilis
[88]		Zwitterionic	*E. coli*
		Antibacterial	*S. aureus*
		Non-hemolytic	
[90]		Zwitterionic Antibacterial	*E. coli*
		Protein adsorption resistant	
[91]		Gemini-QAS	*S. aureus*
		Antibacterial	
[93]		Antibacterial	*E. coli*
			*S. aureus*

Other studies are focused on the incorporation of the QAS moieties through the SS [92], for example by modification of biobased epoxidized soybean oil with dimethylphenylammonium iodine [93]. Obtained PU showed excellent contact-killing properties. A bacteria inhibition zone was even observed with increasing QAS amount in the PU, due to iodine release from iodine QAS counter ion oxidation, which is a highly efficient antimicrobial agent.

Increasing PU hemocompatibility and thromboresistance is another challenge. One approach is developed by blending PU with hemocompatible components such as heparin [94], and another more frequently developed consists of nitric oxide (NO) releasing groups in blood in contact with the materials [2,[95], [96], [97]]. Many catalytic NO-generating materials have been developed and are able to decompose S-nitrosothiols into NO. S-nitrosothiol are molecules found in red blood cells that, when decomposed in NO, send a signal to muscles for vasodilatation and bloodstream increase [98], to avoid thrombosis. Moreover, NO has intrinsic antibacterial properties [96]. However, the catalytic surface of those polymers, usually obtained by coating, lacks NO release stability due to catalytic sites loss via surface erosion over time. To avoid that, a new TPU with a selenium-based chain extender, 2,2′-diselenodiethanol was designed [99]. Organo-selenium have shown catalytic properties for NO obtention from endogenous S-nitrosothiols. Synthesized materials exhibited indeed outstanding catalytic activity with only few amounts of selenium-based chain extender compared to classic diselenide/dopamine coated PUs. Moreover, these new PUs demonstrated excellent NO release stability in PBS for 30 days, as well as higher platelet adhesion resistance, biocompatibility and hemolysis ratio compared to its pristine TPU structure with BD as chain extender. Before that, incorporation of chemically linked catalytic sites as part of the TPU backbone was never described, and the obtained results demonstrated great potential for further development.

### 3.2 Grafted active compound

**Fig. 4:**
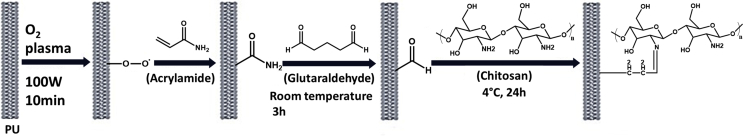
Schematic representation of the process with oxygen plasma treatment followed by chitosan immobilization on PU [110].

Chemical modification of pendant chains is another option to increase PUs bioactivity via grafting [72] or active compound immobilization via surface treatment of biomaterials. In this frame, PU modification usually involves incorporation of active chemical functions in the backbone as a first step, and grafting of bioactive functions as pendent group in a second step [100,101]. These materials also target specific properties as cell proliferation, bactericidal activity and thromboresistance. For instance, chemical grafting of biomimetic pendent groups was performed with heparin-mimicking -O-SO3H (sulfate) or –NH–SO3-H (sulfamate) [102]. Anticoagulation and bacterial adhesion were evaluated, and it was demonstrated that sulfate- and sulfamate pendent chains enhanced antibacterial activity and hemocompatibility, mainly due to an increase of surface hydrophilicity with the high density of -SO3H. Grafted PCL, PPO, PCL [103] and PEG [104] as pendent chains showed enhanced antibacterial activity induced both by steric repulsion and the formation of a physical hydrophilic barrier, especially with PEG brushes. Click-chemistry is a valuable pathway for the preparation of bioactive pendent groups via alkene [105] or alkyne [100] groups either in the SS or in the HS, for instance via thiol-yne addition [106]. Another common surface modification method is based on oxygen plasma treatment for surface activation, followed by bioactive molecules immobilization such as ECM-mimicking biobased collagen [107], anticoagulant biobased heparin [108] or anti-microbial and biobased chitosan [109]. An example of this preparation and process is depicted in Fig. 4. With regard to decreasing PU toxicity concerns due to TDI, as described in 2.2.4, the group used the same formulation but replaced TDI by aliphatic HDI [110]. Antibacterial activity was found less efficient with HDI than the aromatic diisocyanate for a same chitosan concentration, however no explanation was yet found to explain this behavior.

### 3.3 Nanocomposite materials as active compound

Enhancement of PU bioactivity can be also obtained using different types of nanofillers. The most common are the incorporation of nanoparticules (NPs) inside the PU matrix. Desired properties such as antibacterial activity or enhanced mechanical resistance is therefore strongly dependent on their forms and, particles sizes and distribution s [111,112]. For instance, non-uniform distribution or aggregation inside the matrix can lead to poor results [111]. Among the studied NPs types, silica usually exhibit great cytocompatibility enhancement. Silica NPs can be bought or prepared in laboratory, and their common addition is performed by introduction during PU synthesis [113,114]. When preparing silica NP in different pH conditions [114], it was demonstrated that NPs demonstrated better mechanical behavior, but a slightly lower cytocompatibility assay when the synthesis is performed in acidic conditions. Another silica NP type is bioactive glass and it can be synthesized by the Stöber process [115] followed by an ultrasonic –based incorporation [116]. Obtained materials usually exhibit higher mechanical properties and cytotoxicity reduction compared to the corresponding neat PU. Regarding antibacterial properties, silver NPs are largely used due to their excellent antibacterial and bactericidal properties. Despite their cytotoxicity, in low concentration they are found non-toxic in vitro [117]. Moreover, silver NPs prepared by in situ reduction of silver ion, usually in form of silver nitrate [118,119], to particles results in better NP distribution homogeneity inside PU matrix [111,117,120]. For instance, anionic WPU loaded with silver nitrate shows enhanced mechanical properties and outstanding antibacterial activity against E. colis with a bacterial reduction of 99.99% [121]. The same material also exhibited 54% bacterial reduction against Gram-positive bacteria (*S. aureus*). As mentioned earlier, NPs shape plays a crucial role in material bioactivity [122]. For instance, spherical and triangular shaped Ag Np were prepared in situ in a biobased WPU matrix [123]. Antibacterial properties were found significantly higher against both *E. coli* and *S. aureus* with the triangular shaped particles, higher activity being due to a higher amount of reactive (1 1 1) crystal faces, shown by XRD.

## 4 Conventional (fossil-based) PU for biomedical applications

As previously mentioned, different conventional (i.e., fossil-based) PUs have been developed for biomedical purposes since decades due to their tunable properties, low density and cost-efficiency compared to classical biomaterials such as metals or ceramics. To be used as medical device, PU structure, HS/SS ratio, type and form should meet the desired application requirements. In this regard, a careful choice of the different compounds and quantities for PU formulation should be done (polyol, polyisocyanate, chain extender).

### 4.1 Non implantable devices

For applications such as wound dressings where the material is meant to be removed after usage, some commercial fossil-based PU foams are already available on the market such as for example Medifoam®, PermaFoam® or Suprasorb® [124]. Many studies revealed the potential of polyether-based PU for this application due to the flexibility, the great hydrophilicity and the water uptake capacity for exudates absorption bring by the polyethers in the formulations. Thus, most PU wound dressing materials are based on different polyether types and molar masses like polyethylene glycol (PEG) [125], polypropylene glycol (PPG) [126] or a mixture of both [127] for a tailored water absorption. Recent studies are focussed on conferring PU enhanced properties via the incorporation of bioactive compounds. Many properties with sight of a better wound healing process have been obtained such as hemostasis [128,129], antimicrobial [126,127] with cell proliferation inducing effects like electroactivity [130] and with silica NPs [131], or finally comprising growth factors for targeted patient's condition such as diabetes [132]. It is indeed known that diabetes wounds endure a slower healing process than other wounds due to many factors such as a delay in collagen synthesis, but the precise role of most of them are not yet fully understood [133]. Improved water absorption was also obtained from other 3D-network PU like waterborne PU hydrogels [134].

### 4.2 Long-term implants

Scaffolds preparation is one of the main PU applications for long-term implantable biomaterials due to the versatility of their mechanical and structural properties, and their inherent biocompatibility. Indeed, PU can be prepared as porous networks mimicking soft or hard tissues and engineered for enhanced cell adhesion, proliferation and differentiation. This latter is based on the ability of mesenchymal stem cells (MSCs) to differentiate into a specific tissue-cell type. For hard tissues applications where a strong mechanical support is needed, highly crosslinked networks [135], robust aromatic HS [136,137] and PU-urea [138] structures are usually designed. For enhanced mechanical properties, bioactive composites using nanoclays [139], Bioglass® (silicate bioactive glass) [140] and hydroxyapatite (HA) [137,138] have also been developed. Besides, nanoclays and HA also trigger cell adhesion and osteogenic (bone tissue cells) differentiation. In the soft tissue area and in contact to hard tissues, flexibility is a key point for a successful integration as well as cell adhesion and proliferation. For that, long and flexible SS based on polyester polycaprolactone (PCL) diol [141,142], polycarbonate (PC) diol [[143], [144], [145]] and/or polyether like PEG or polytetramethylene glycol (PTMG) diol structures have been extensively studied for non-specific [[146], [147], [148]] or specific soft tissue mimicking such as cardiac applications [[149], [150], [151]], cartilage [152], nerve conduits [[153], [154], [155]] or vessels [[156], [157], [158], [159]].

### 4.3 Short-term implants

For areas where degradation should be tunable like drug delivery, PUs are usually developed in forms of electrospun networks [160,161], hydrogels [162,163], membranes [164] or nanocarriers like micelles [165] when quick drug release is needed. As PUs degradation process is mainly determined by the SS, degradation rates for tailored drug release time are obtained by varying the type and amount of polyols undergoing hydrolytic and enzymatic degradations such as polyesters-based polyols, to hydrophilic polyols undergoing water uptake and oxidative degradation like polyether-based polyols [166,167]. Tunable degradation for specific applications such as for example, cancer treatment was designed by incorporation of various stimuli-responsive bonds in the SS or HS of the PU architectures or molecules. We can find for instance imine (C=N) or sodium bicarbonate (NaHCO3) [168,169], disulfide (S–S) [[170], [171], [172]], and selenium-inserted polymers [173], triggered by pH, redox or light, respectively. In this case, the degradation rate and drug release depend on the type and amount of stimuli-responsive systems in the PU backbone.

### 4.4 The specific case of PUs as vascular occlusion devices

Due to genetical heritage, hypertension, malformation and many other diseases, aneurysms can form at blood vessel walls, especially at the division point where smaller blood vessels form. Aneurysms are in form of bulge that grow via blood filling and mainly cause thinning and weakening of the affected vessel. The main risk following the formation of aneurysm is they rupture which causes intern hemorrhage leading in the worst case to patient's death [174]. Endovascular embolizations are performed to prevent aneurysm rupture and rely on a minimal non-surgical technique that occludes the aneurysm or deviates the blood flow to another region. The process consists of an embolic agent introduction via a catheter or micro-catheter depending on the affected blood-vessel, filling the aneurysm and preventing its growth. Current procedures use coils, mesh and foams, metal coiling (especially platinum) being today the reference material [175]. As an improved alternative solution, shape-memory polymers (SMP) have been developed for better occlusion, occlusion-rate and healing. SMPs are composed of moieties able to respond to one or several external stimuli. This property has been used to develop PU-based SMPs (SMPUs) with a fixed temporary shape that, once external stimulus is applied, recovers the original shape. For vascular occlusion devices, SMPUs have been synthesized to answer to temperature [176] and/or pH stimulus to be inserted in a temporary form via catheter or micro-catheter and recover the original shape at blood-vessel temperature (~37 °C) or pH (~7.4).

#### Temperature responsive SMPUs

In the last years, Maitland and coworkers have developed, after a long-standing work on the subject, SMPUs foams [176] with multiple properties such as radio-opacity [177], anti-oxidation [178,179] and with tunable mechanical properties [179,180]. All results led to in vivo implantation studies with both porcine sidewall aneurysm model [181] and rabbit elastase model [182], compared to standard bare platinum tungsten coil. Great healing improvements were observed with SMPU foams. More recent studies concentrated on monomeric HDI substitution by tris-(2-hydroxyethyl) isocyanurate [178]. Moreover, these works have inspired other research groups for the preparation of embolic devices [183,184].

#### pH responsive SMPUs

**Fig. 5:**
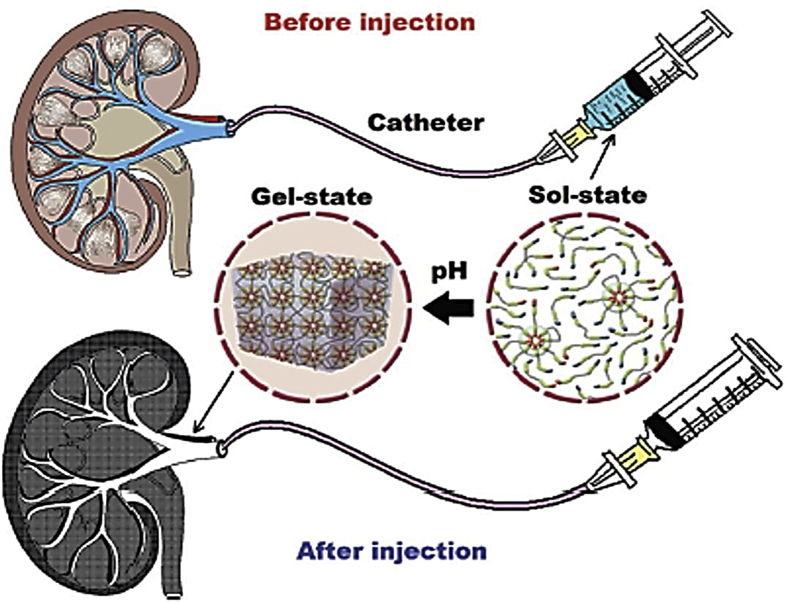
Schematic representation of a pH-responsive SMPUs as embolic agent [185].

As previously mentioned, SMPUs can also recover their shape after being exposed to blood-vessel pH. In this purpose, sulfamethazine-based hydrogel SMPUs have been synthesized as injectable embolic agent [185,186] with radio-opacity properties. After a quick sol-gel process at body pH, a gel is obtained that occludes the aneurysm, as depicted in Fig. 5. The hydrogel was found stable and efficient for renal artery in vivo in rat models even after 12 weeks, and MTT assay on cells derived from kidney cells combined with in vivo results exhibited great biocompatibility, demonstrating a potential candidate for arteries embolic agent.

## 5 Biobased PU for biomedical applications

### 5.1 Generalities

**Fig. 6:**
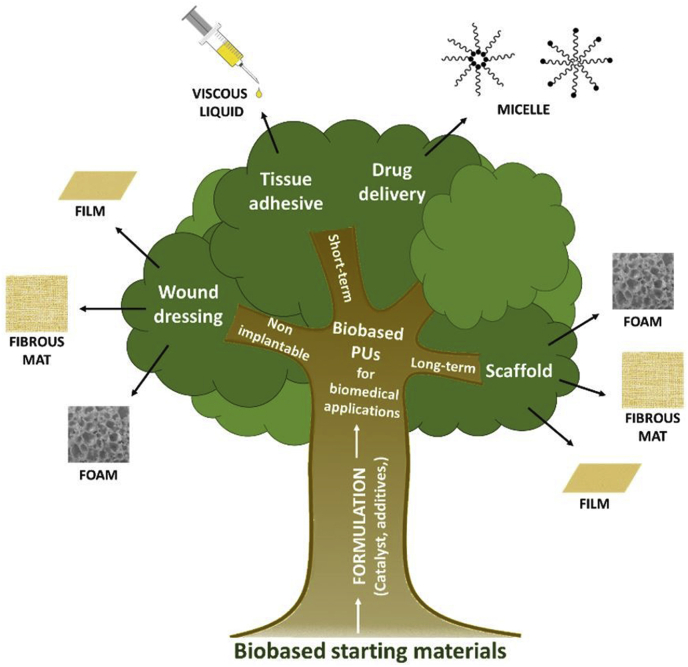
Main biobased PU biomedical applications and their corresponding structures.

In the last decades, growing interest in the use of renewable materials for a large range of application has led to the emergence of new building blocks and new chemical architectures for PU synthesis. The goal can be (i) to replace and copy existing fossil-based molecules like polyfunctional molecules as glycerol, BDO or 1,3-propanediol [187] or (ii) for the elaboration of new macromolecular architectures from several emerging biobased building blocks obtained from biomass, usually obtained by combining chemistry and white biotechnology processes in a chem-biotech approach, as described earlier in part 2.2.3. Since some decades and by biotechnology, several new building blocks can be largely available such as some bacterial polymers or oligomers, furans or isohexides. As emerging technology and on agreement with some principles for a green chemistry, biobased PUs are slowly replacing fossil-based ones in many applications with an environmental gain in term of Life Cycle Analysis (LCA) [188] in form of for instance, foams [189] membranes, or coating. The corresponding researches are mainly focused on developing and using these novative PU architectures (i) to replace conventional PUs, and/or (ii) to develop new biomaterials with enhanced properties, for the biomedical area. The chemical structure of the biobased PU, as well as its final form (membrane, foam, hydrogels) and shape must be adapted according to the targeted biomed application (for example implantable or non-implantable systems), as depicted in Fig. 6.

### 5.2 Non implantable devices

#### 5.2.1 Wound dressing

Skin acts as the most important barrier for protecting organs from external attacks. Therefore, when the skin is damaged, a wound healing process starts to replace the damaged tissues with four main stages: hemostasis (in case of bleeding), inflammation, proliferation and maturation, as depicted in Fig. 1. However, depending on the wound type, depth, origin and infection level, as well as the patient's condition (diabetes, cancer), skin repair takes at least few days and up to months in worst cases. Chronic wound care standard consists of wound debridement (removal of necrotic or infected tissues), followed by swabbing for infection, cleaning, and finally dressing using an adequate material [190]. This step is particularly sensitive, a non-appropriate material being the potential cause of wound drying or over-moist and further bacterial infection, especially since the emergence of antibiotic-resistant bacterial strains such as *S. aureus* and *P. aeruginosa* [191]. Consequently, the development of adequate wound dressings has become essential, not only to prevent previously mentioned infections, but also to provide a right environment for wound healing. By right environment is understood a material that promotes oxygen penetration and adequate moisture while absorbing exudates, as well as limited adherence to avoid skin re-trauma after dressing removal [192]. Biopolymers and more generally biobased compounds have been used since decades for the preparation of wound dressing materials due to their inherent biocompatibility, hydrophilicity and swelling capacity. Among them, polysaccharides like cellulose [193] and derivates, chitin, chitosan [194] or alginate [195] have proven to be efficient for regenerative medicine, mainly as hydrogels. With the emergence of new biobased materials for biomedical application, it was evident that the incorporation of biobased moieties such as vegetable oils, polysaccharides, lignin-derivatives, into PU matrix could have specific effects in terms of degradability and could bring new properties.

Recent developments on biobased PUs for wound dressing applications in form of foams, porous fibrous mat or films are multiple and are summarized in Table 4.

**Table 4:** Main properties of recent PU studies for wound dressing applications.

Ref	Form	Main reagents	Catalyst	Properties + Agent used	Pore size (μm) or fiber diam (nm)	Young's modulus	Wound healing after 14 days	Biobased
[127]	Foam	EOPO	none	Antibacterial	<100 μm	n.m		No
		PEG		Wound healing				
		Glycerol						
		TDI		Silver-HA NPs				
[126]	Foam	PPG	DBTL	Antibacterial	n.m	n.m	n.m	No
		TDI		Endotoxin adsorption				
		Imidazolium diol		Cationic Foam				
[132]	Foam	EOPO	none	Diabetic wound adapted	<200 μm	n.m		No
		PEG						
		Glycerol		rhEGF				
		TDI						
[131]	Foam		None	Mechanical properties	450–500 μm	500–1300 kPa		No
		HYPOL2002		Wound healing				
		Glycerol						
		TDI		Silica NPs				
[128]	Foam	PEG diol Glycerol ethoxylate Pluronic F-127 HDI	DBTL	Hemostatic	500–1000 μm	458–705 kPa		No
			KKAT XK-672	Kaolin			n.m	
[129]	Foam		Stannous octoate	Hemostatic Wound healing	50–300 μm	n.m-675 kPa		No
		PEG						
		PACM						
		HMDI						

[130]	Film	PCL PEG HDI AT HDA	Stannous octoate	Non-adherent Electroactive SMP Antioxydant Quick drug release Biodegradable	Elastomer	9.1–12.9 MPa		No
[196]	Foam	PEG Glycerin Na-alginate HDI Alginate hydrogel Jute fibers	DBTL	Mechanical properties	500–600 μm	0.785–2.987 kPa	n.m	Yes
				Drug release				
				Super-absorptive				
[197,198]	Foam	PPG	Tin T9	Antibacterial Drug release Silver NPs Asiaticoside	228–318 μm	10.7–15.3 kPa		Yes
		Dabco DC5810						
		Dabco 33-LV						
		TDI						
		HPMC						
		Chitosan						
		Na-alginate						
[199]	Foam	PEG	DABCO	Antibacterial Lignin-encapsulated silver NPs	n.m	55–78 kPa	n.m	Yes
		Glycerol						
		DC 5179						
		MDI						
[200]	Foam	POLIOS 55/20	DBTL	Antiseptic Antibacterial Cinnamaldehyde	65–426 μm	244–254 kPa	n.m	Yes
		HMDI						
		BDO						
[201]	Foam	POLIOS 55/20 HMDI BDO PLA	DBTL	Antibacterial Fast-degradable Ciprofloxacin	50–375 μm	n.m	n.m	Yes
[203]	Foam	Dimeric fatty acid polyol MDI BDO	none	Antibacterial ZnO NPs	10–60 μm	n.m	n.m	Yes
[204]	Fibers	PCL MDI Ethanediamine Chitosan Gelatin	none	Antibacterial	232–287 nm	n.m	n.m	Yes
				Hemostatic				
				SMPU				

				Silver NPs				
[205]	Fibers	PCL MDI BIN	none	Antibacterial	245–727 nm	n.m	n.m	Yes
				Hemostatic				
				SMPU				
[206]	Fibers	PCN	DBTL	Mechanical strength	2 μm	100–400 kPa	n.m	Yes
		HDI		Tunable degradation				
		BDO		Wound healing				
		Gelatin						
[207]	Fibers	CC-ME	TBD	Isocyanate-free Water uptake	0.72–4.6 μm	0.9–109 MPa	n.m	Yes
		1,12-Diaminododecane						
		Jeffamine THF-100						
		PTMO						
[211]	Film	CO or CAPA 7201 HDI Diethylene glycol	none	New structures	none	n.m	n.m	Yes
[212]	Film	CO or ricinoleic methyl ester	DBTL	Electroactive Antibacterial Antioxydant	None	n.m		Yes
		IPDI						
		APTMS						
[214]	Film	CO	DBTL	Partially NIPU	none	n.m		Yes
		ESBO		Antibacterial				
		DMDEA						
		IPDI		QAS				
[215]	Film	CO	none	Antibacterial Oxygen plasma treatment	none	n.m	n.m	Yes
		Desmodur N75						
		HDI						

EOPO: Ethylene Oxyde/Propylene oxide copolymer; PACM: 4,4′-diaminodicyclohexylmathane; HPMC: hydroxypropyl methylcellulose; BIN: N,N-bis(2-hydroxyethyl)-isonicotinamide; PCN: poly(1,6-hexyl 1,2-ethyl carbonate); CC-ME: 9,10-cyclic carbonate-methyl decanoate; ESBO: Epoxidized soybean oil; DMDEA: N,N-dimethylethylenediamine; APTMS: (3-Aminopropyl)trimethoxysilane; n.m: not mentioned; U: unmodified; B: best.

#### Foams as wound dressing materials

PU foams for wound dressing applications have several advantages. With their tunable porosity and pore sizes, they can offer adequate moisture and gas permeability while having a low adherence to the wound site. Successful integration of biobased building blocks like alginate [196] or other polysaccharides trough their hydroxyl groups (OH) in PU foams networks have been obtained for enhanced biocompatibility and kinetic of degradation [197]. Bioactive properties were further added via two agents: silver NPs for antibacterial activity and asiaticoside, a proven herbal wound healing agent [198]. High polysaccharide contents resulted in a powdery surface and decreased tensile properties, thus foams with middle polysaccharide contents were evaluated for their clinical efficiency in human patients with traumatic abrasion or dermal burn wounds [197]. Results indicated accelerated and non-infected wound healing compared to conventional treatments. Silver NPs exhibiting cytotoxicity at high concentrations [111], a way to overcome this issue is by NPs encapsulation with phenolated lignin before being incorporated into the PU foam [199]. With this approach, the foams demonstrated decreasing pore sizes with increasing biobased content, due both to enhanced chemical crosslinking from the lignin reaction, through their OH reaction with –NCO, and physical crosslinking from corresponding H-bonds and π stacking. Other antibacterial porous PU systems were obtained via the salt-leaching method with cinnamaldehyde as antibacterial agent [200], or blended with PLA and ciprofloxacin as antibacterial agent [201,202]. Nothing but biobased polyols as macropolyols for the synthesis of foam-based wound dressings is quite rare. However, a polyester-polyol derived from rapeseed oil was used with MDI and BDO [203]. In this work, Zinc oxide (ZnO) NPs were used as bioactive agent with high antimicrobial activity and no cytotoxicity up to 5 wt% ZnO NPs.

#### Fibrous mat for wound dressing applications

**Fig. 7:**
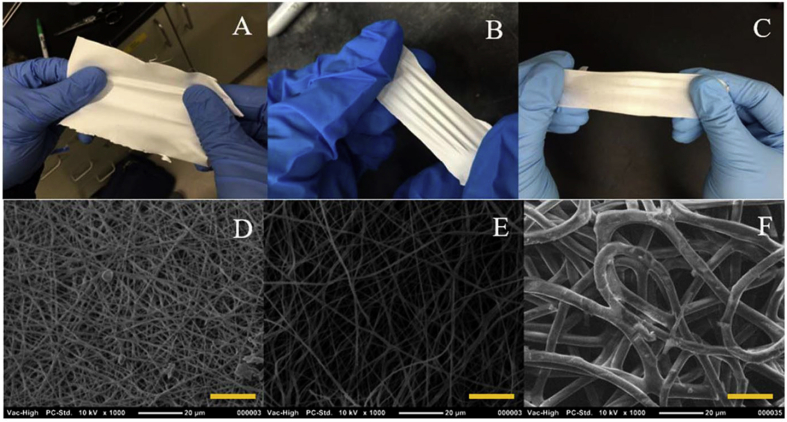
A and B Macroscale images of plant-oil based NIPU fibrous mat, with increasing PTMO amount. C. Macroscale image of the TPU control. D and E, SEM images of plant-oil based NIPU fibrous mat, with increasing PTMO amount. E, SEM image of the TPU control. Scale bars: 20 μm [207].

Electrospinning has now become a conventional method for the elaboration of wound dressing materials [192]. Better properties such as hydrophilicity, biocompatibility and flexibility were achieved by blending TPUs with biopolymers before electrospinning, such as gelatin and/or chitosan [204]. Another technique is based on the formation of a bilayered mat composed of electrospun TPU followed by gelatin electrospinning [205]. For instance, TPU-gelatin-chitosan blends with antibacterial properties were obtained with AgNO3. In this case, PU was the mechanical support and gelatin/chitosan were additional components. As expected, the nanofibers exhibited better hydrophilicity than the neat PU by the incorporation of hydrophilic chitosan and gelatin in the PU matrix. Furthermore, significant antibacterial properties were found, coupled with in vitro cytocompatibility and a better hemostatic effect compared to neat PU. In another study, gelatin was used as matrix and the PU as the additional component to improve the mechanical properties [206]. Gelatin/TPU blends used in vitro and in vivo testing in mouse model demonstrated great biocompatibility and comparable cell migration to neat gelatin, as well as a progressive degradation over time. Neat gelatin was fully degraded in 3 h against more than 7 days for the blend. More recently, NIPU fibers were also successfully electrospun into a fibrous mat [207], from which the obtained materials are presented in Fig. 7. A and B Macroscale images of plant-oil based NIPU fibrous mat, with increasing PTMO amount. C. Macroscale image of the TPU control. D and E, SEM images of plant-oil based NIPU fibrous mat, with increasing PTMO amount. E, SEM image of the TPU control. Fig. 7. NIPU synthesis was previously reported [208] and based on cyclic carbonate aminolysis of a methyl 9-decenoate cyclocarbonate derivate (mostly present in palm or coconut oil) using an organocatalyst, 1,5,7-triazabicyclo [4.4.0]dec-5-ene (TBD). In vitro cytocompatibility, higher Young's modulus and better water uptake than a classic poly (tetramethylene) oxide (PTMO)-based TPU fibers were obtained, which are key parameters for wound dressing applications.

#### Films as wound dressing materials

Biobased membranes have also been elaborated for wound dressing applications, several of them are based on vegetable oils [[209], [210], [211]]. For instance, hybrids PU/siloxanes from methoxysilane (Si–OMe)-terminated castor oil (CO)-prepolymer and a ricinoleic methyl ester-based prepolymer were prepared [212]. Electroactivity, significant antioxidant and antibacterial properties were obtained via the incorporation of a synthesized aniline tetramer [213]. In vivo wound closure evaluation on a full-thickness skin wound was performed. The membranes containing aniline tetramer moieties demonstrated the best wound healing after 20 days with higher (100%) wound closure compared to membranes without aniline tetramer. In an effort to develop more environmentally-friendly processes, another partially NIPU antibacterial membrane was recently developed from epoxidized soybean oil [214]. The epoxidized fatty acid was first modified into a cyclocarbonate soybean oil. Then, through aminolysis and further treatment with iodomethane, a QAS polyol was obtained and used with CO and IPDI for the elaboration of membranes. They presented high in vitro and in vivo antimicrobial activities against bacteria collected on rat models wound beds, and with excellent cytocompatibility and in vivo wound healing comparable to the gauze. In another study, CO was reacted with HDI trimer to form a crosslinked PU [215] and further surface modified with a low-pressure plasma treatment, followed by chitosan or collagen immobilization. Both grafted PU exhibited high fibroblast cells proliferation and viability, hydrophilicity and antibacterial activity. For all tested properties, chitosan-grafted membrane always exhibited better results than gelatin-grafted PU, except with Gram positive bacteria where collagen demonstrated better antimicrobial activity due to electrostatic interactions between the positive charges on the bacteria wall and the negative charges of the carboxylate groups from the collagen.

### 5.3 Long term implants

#### 5.3.1 Scaffold

The various human body tissues display a large range of properties and structures. Tissue engineering is based on the cell culturing using scaffolds for further incorporation into the body. This technique aims to accelerate tissue reconstruction after traumatic experiences for tissues such as for instance surgery. Tissue scaffolds generally need porous materials presenting interconnected pores with sizes from the nano to the microscale. Engineered scaffolds tend to mimic tissue microstructure and mechanical properties for enhanced cell proliferation and differentiation. Most discussed tissues and the adequate scaffold properties are summarized in Table 5. Contrary to soft tissue, hard tissue refers to tissues with high elastic modulus and low flexibility like bone tissues. Along with the conventional foaming process, TPU-based porous scaffolds can be obtained via different techniques from which the main ones are solvent casting/particle leaching, thermally induced phase separation and emulsion freeze-drying [216].

**Table 5:** Main required scaffold properties.

Application	Adequate modulus	Adequate cells	Adequate porosity	Adequate
				pore sizes range
Cancellous bone	1.28–1.97 GPa [217]	Osteoblast	75–90% [218]	140–600 μm [218]
		Osteocyte		
Cortical bone	10.4–20.7 GPa [217]	Osteoblast	5–10% [218]	10–50 μm [218]
		Osteocyte		
Skeletal muscle	5–170 kPa [217]	C2C12 [143]	around 90% [143]	50–200 μm [220]
		SMCs [219]		
Vein	34 kPa (circumferential) [221] 102 kPa (longitudinal) [221]	Endothelial	n.m	6,5–7,6 nm [158]
Aorta	128 kPa (young) [221] 41.7 kPa (old) [221]	Endothelial	n.m	1–20 μm [222]
Nerve guide [223]	0.30–30 MPa	Neuro 2a	60–80%	30–50 μm
		Glial cell		
		Schwann cell		
Ear cartilage [224]	5 MPa	Chondrocyte Osteocyte	n.m	>100 μm
Cartilage	2.8–18.6 MPa [152]	Chondrocyte Osteocyte	75–87% [225]	75 μm–175μm [225]
Cardiac tissues	5–50 kPa [217]	Cardiomyocytes H9C2 [226] or	75%–96% [228,229]	Some-300μm [229]
		HL-1 [227]		

n.m: not mentioned; SMCs: Smooth Muscle Cell.

##### 5.3.1.1 Hard tissues

**Fig. 8:**
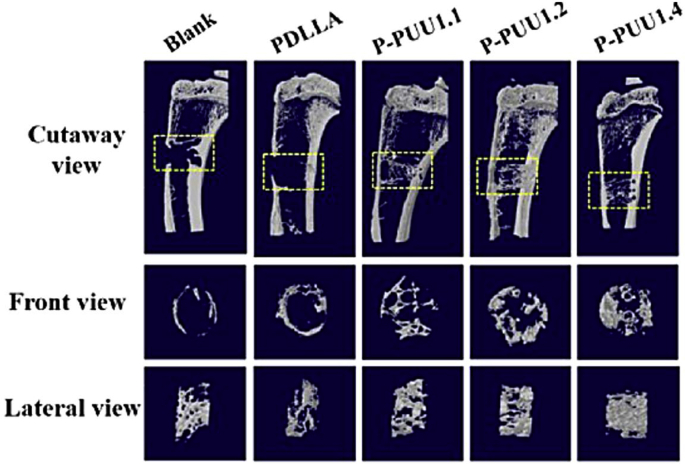
Micro-CT analysis of new bone formation at 8 weeks post-implantation: 3D reconstruction model of new bone formation, PUs with increasing piperazine content from P-PUU1.1 to P-PUU1.3 [237].

Bone tissue regeneration based on PU scaffolds can lead to certain disadvantages such as poor cellular adhesion, poor differentiation and biomineralization even if the scaffold physico-chemical and mechanical properties are adequate for bone tissue growth. This can be linked to some degradation products which causes significant pH changes in scaffold microenvironment. It has indeed been reported that osteoblast proliferation and differentiation is promoted for a pH around 7.40, close to the physiological value [230]. The general lack of bioactivity of PUs regarding specific tissue applications such as bone tissue reconstruction however remains another drawback. As mentioned earlier, electroactivity for enhanced osteogenic differentiation can been achieved by aniline trimer (AT) incorporation [231]. Bioactive NPs such as HA have also been incorporated into biobased PU's matrix for enhanced biocompatibility and differentiation [232]. 40 wt% HA in a fully biobased foam with CO, IPDI and BDO [233] was suitable for short term calcium phosphate deposition, demonstration a higher biomineralization and found adequate for bone tissue regeneration due to adequate porosity and pore sizes of 78–81% and 300–1000 μm, respectively (Table 5). For enhanced mechanical biomimetic, they further modified CO by alcoholisis with glycerol [234] for PU architecture. It was demonstrated that the scaffold with glycerol-modified CO had outstanding mechanical properties due to a better elastic modulus up to 165.36 MPa, possibly thanks to less dangling and unreacted end-groups than CO-based scaffold. A great bone matrix and trabecula regeneration were obtained, as well as excellent biocompatibility and osteogenic differentiation. Another technique is based on bone ECM-component grafting to increase cell affinity and consequently promote a high bone tissue regeneration [235]. For that, fatty acid-based polyols were prepared using alkyne modification, followed by thiol-yne coupling as previously discussed [236]. TPUs from these polyols were then surface modified by aminolysis and activated with HCl to allow further ionic immobilization of the polysaccharide salt chondroitin sulfate sodium (CS), a bone ECM component that promotes bone cell (osteoblast) adhesion. Better surface hydrophilicity and a significant increase in osteoblast cell viability were obtained. It was concluded that the prepared scaffold was suitable for cell growth. To avoid pH changes in the bone microenvironment during scaffold degradation, SMP TPU-urea were synthesized using poly (D,l-lactid acid) (PDLLA) diol with piperazine moieties [237], from which the synthesis was previously described [238]. HDI-isosorbide-HDI [239] or HDI were used as diisocyanates. Scaffolds were further obtained by air-driven extrusion 3D-printing using a predesigned 3D structure model [237]. Compressive modulus increased with the piperazine content (0.1–0.4 molar equivalents) from 131 up to 156 MPa, respectively. The scaffolds exhibited excellent cytocompatibility coupled with significant new bone formation after 8 weeks in vivo implantation, as depicted in Fig. 8. These higher properties were explained by a stable neutral pH over time via PDLLA acidic degradation products neutralization by piperazine [240,241]. They also demonstrated that cell behavior regulation can potentially be achieved by SMP stretching-induced nanostructure [242].

##### 5.3.1.2 Soft tissues

Soft tissue reconstruction is a domain where several biobased PU systems have been investigated for a large range of applications such as cartilage, muscle, cardiac systems, vessel, nerves or muscles.

###### Cartilage

**Fig. 9:**
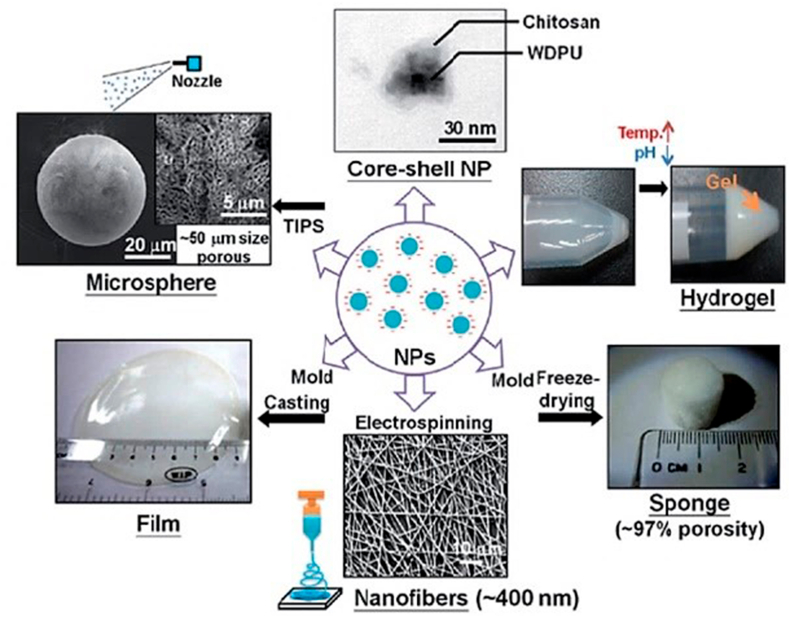
Processing based on the NPs self-assembly [249].

Cartilage tissues, unlike other soft tissues, need higher mechanical strength and modulus. PU scaffolds have been elaborated for this application using supramolecular ionic bonds from alginate-based PU elastomers with tunable high mechanical performance [243]. Young's moduli were ranging from 14 to 93 MPa. Scaffolds exhibited slow degradation rate in vivo, which was comparable to PCL-based scaffolds. The synthesis of water-based PU micelles [244] has also been tested with the synthesis of biodegradable PU in form of ionic NPs or porous scaffold [245], as depicted in Fig. 9. Scaffold was then prepared by 3D-printing in a fibrous mat using polyethylene oxide as viscosity enhancer [244]. Waterborne PU indeed possess a too low viscosity to be 3D-printed without modifications [246]. After preliminary and encouraging scaffold properties, researchers went further by loading chondrogenic induction factors and blended the PU with hyaluronan solution for enhanced cartilage repair [247]. Controlled drug release and increased bioactivity were observed as MSCs cells successfully underwent chondrogenesis (cartilage tissue development). In a recent study, induction factor-loaded PU microspheres have been developed followed by 3D-printing [248]. Results depicted a sequential drug release which was adapted to chondrocytes cell growth. Studies on these waterborne PU ionic NPs led to the formation of platform PUs with tunable processes for many types of applications, as depicted in Fig. 9 [249].

###### Muscle

Biobased TPU films mainly based on biobased polyester such as PLA [250] or PHA derivates were recently developed for muscle applications. In a recent study, new PUs from PLA diol, poly (3-hydroxybutyrate-*co*-4-hydroxybutyrate) (P3/4 H B) diol and HMDI were developed [219]. Two TPUs were prepared, an altering P3/4 H B and PLA blocks called PULA-alt-3/4 H B, and a random PLA and P3/4 H B distribution, called PULA-ran-3/4 H B, respectively. These systems were compared to equivalent systems based on P3/4 H B and PCL, from a previous study [251]. Porous scaffolds from PULA-alt-3/4 H B expressed better shape recovery, as well as hemocompatibility, in vitro and in vivo cytocompatibility and hydrophilicity due to a more regular structure compared to the random block polymerization, which makes it a more suitable candidate for muscle tissue scaffolds. 6 weeks implantation in rats was successful and no inflammation coupled with formation of muscle tissues was observed for the alternated and random scaffold structures.

###### Cardiac system

Studies for cardiac system applications are mainly focused on the preparation of biomimetic cardiac patch with elastomeric properties while maintaining mechanical support for the tissue. Current demand in biocompatible and bioactive cardiac patch for enhanced cell adhesion and proliferation is rising due to a lack of efficient solutions. Most biodegradable polymer scaffolds, comprising last trends in PU-based patch, have recently been reviewed [252]. Many cardiac patch or cardiac scaffold are PCL-based PUs and comprise urethane as well as urea groups for improved mechanical properties [228,253]. However, biobased alternatives are nowadays explored using for instance vegetable oil-based polyols such as CO [227]. Electroactivity was added by incorporation of increasing oligomeric aniline moieties amounts [254] in order to favor cell proliferation. In this case, the obtained scaffolds exhibited increasing conductivity with aniline content. However, degradation in PBS was slow and only the incorporation of medium content of aniline showed adequate cell cytocompatibility. Successful electrical stimulation of cardiomyocyte cells for enhanced adhesion, proliferation and further cardiac tissue regeneration was also obtained by incorporation of synthesized gold nanotubes/nanowires in a CO-based PU [255]. m.

###### Vessel

Currently, just as for hard tissue repair, most dominant procedure for vessel replacement is by autogenous graft. However, some patients lack adequate autogenous tissues, leading to the use of synthetic vascular grafts. Large diameter conduit (>6 mm) grafts already have efficient and reliable solutions. There is nonetheless a need for the development of small vessels scaffold for enhanced compatibility and increased tissue regeneration [158]. Biobased PU scaffolds have been extensively studied for vascular applications in the last years due to their specific mechanical properties, biocompatibility and tunable degradability. Various scaffolds forms are described, from films [256,257] to porous foam [258,259] or electrospun fibrous mat [260]. For instance, biobased PUs networks were prepared with a lysine triisocyanate PEG prepolymer with a previously synthesized polyester triol from glycerol, ε-caprolactone, D,L lactide and glycolide [261,262]. Porous structures with 77–84% porosity and 250–290 μm pore sizes were obtained with sucrose leaching method. Injectable solution of the prepolymer, sucrose NPs and the polyester triol was prepared and administrated to a porcine model [261] with promising results, but with need for further optimization despite noteworthy setting time, cell proliferation and collagen accumulation. A previous study was already performed on rat models using hyaluronic acid or carboxymethylcellulose instead of sucrose [262], with high biocompatibility and comparable setting times than with sucrose as porogen agent. PU films have also been prepared from PCL diol, poly (3-hydroxybutyrate-*co*-3-hydroxyvalerate) (PHBV) diol and MDI [263], with up to 60 mol% PHBV. Flexible films were obtained with tunable mechanical properties: Higher PHBV content in the PUs gave higher Young's moduli from 0.3 to 20.6 MPa for 30 and 60 mol% PHBV diol, respectively. PHBV incorporation also increased surface hydrophilicity of the film, as well as cytocompatibility with MSCs and outstanding hemocompatibility with low hemolysis and platelet adhesion. These films were thus concluded to be good candidates for blood-contacting implants such as vascular grafts. Electrospun drug-loaded SMPU nanofibrous mats from PCL diol mixed either with PLLA diol and PEG (PU-PLLA/PEG) or poly (lactic-*co*-glycolic acid) diol (PLGA) (PU-PLGA) were prepared [260] and exhibited controlled rapamycin release in PBS for 9 months at 37 °C. Rapamycin is an immunosuppressant drug for enhancing biocompatibility and/or prevent implant or organ transplant body rejection. Despite noteworthy in vitro performance in drug release, shape recovery and in vitro cytocompatibility, further investigation regarding in vivo mechanical properties, degradation and biocompatibility must be established.

###### Nerves

**Fig. 10:**
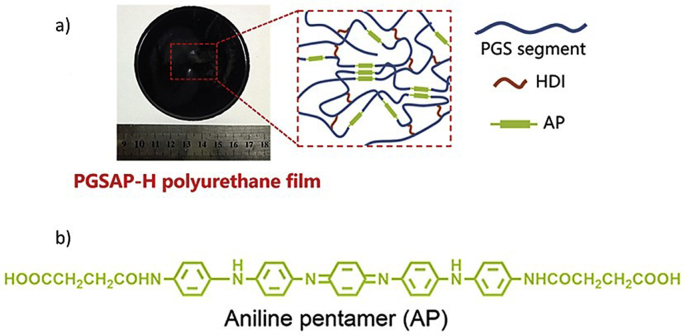
a) Picture and presentation of PU film structure from PGS, HDI and AP and b) electroactive AP structure [154].

The most common clinical disease for the nervous system is peripherical nerve injuries. Current treatments for peripherical nerve injuries is end-to-end connections if the gap is bellow 2–3 cm, and autologous graft for longer gap. To replace autologous grafts for many reasons mentioned earlier, biodegradable and biobased nerve conduit scaffolds have been elaborated to guide the nerve's reconstruction through a tubular graft. For that, PUs have proven to be competitive materials due to their high biocompatibility in addition to tunable mechanical strength, flexibility and biodegradability. Recent studies aim at developing quicker and fully functional nerve reconstruction compared to commercially available products such as for example PGA-based Neurotube©. For that, WPU films and porous PU network were prepared [264] from a formulation previously described and used for several biomedical applications [245,248,249]. Porous PU scaffold exhibited pore interconnectivity and uneven pore sizes for the outer, inner surfaces (42 and 9 μm, respectively) and cross-section (23 μm), respectively. In a previous work [265], asymmetrical pores have demonstrated efficient for wound inflammation waste drainage in the early nerve regeneration stage, compared to symmetrical pores, leading to quicker nerve regeneration. Compared to PU films, porous networks also demonstrated better permeability of model bovine serum albumin (BSA) solution [264], which should demonstrate efficient mass transport for enhanced nerve repair. These PUs were further implanted in rat models for 6 weeks. Compared to Neurotube©, PU porous scaffold exhibited slower degradation, leading to a better nerve support and higher nerve regeneration. New nerve conduits have also been prepared with biobased PU films from poly (glycerol sebacate) (PGS) and aniline pentamer (AP) moieties for electroactivity properties [154] as depicted in Fig. 10. PUs with 15 wt% aniline pentamer incorporation showed the slowest degradation rate combined with significantly higher nerve growth factor (NGF) release from neuronal Schwann cells. Its higher electroactivity also induced neurite growth and elongation. Schwann cells alignment and induced elongation was achieved later by preparing films with AP on PDMS micropattern models [266]. Outstanding increase of neurite elongation was observed, as well as enhanced rat Schwann cell alignment with increased NGF secretion compared to the non-micropatterned scaffold. Combining bioactivity and the micropatterning technique exhibited excellent results for potential future applications as nerve guidance conduit.

### 5.4 Short term implants

#### 5.4.1 Drug delivery

**Fig. 11:**
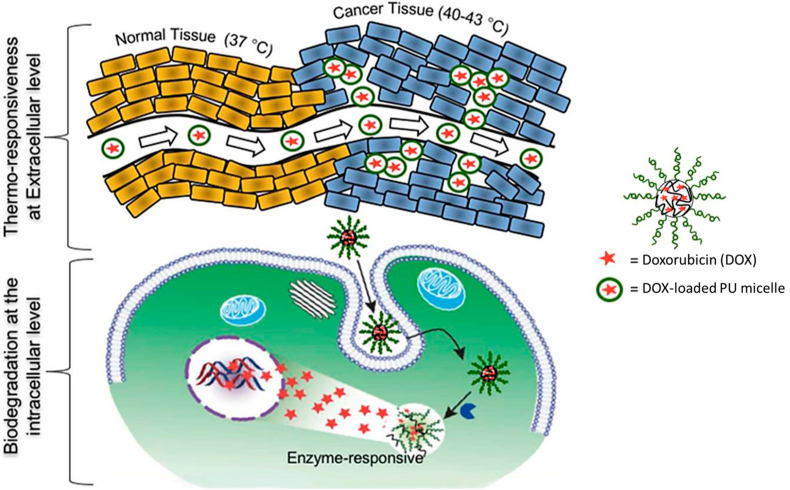
Overall concept of PU micelles bi-responsivity for anticancer drug release, adapted from Ref. [275].

Due to their tunable composition, PUs have been common choices for the preparation of drug delivery devices. The stimuli responsive architectures in PU systems has led to extensive research for specific targeted delivery of loaded drugs for several kinds of applications. The insertion of pH-sensitive or temperature-sensitive polymers can trigger drug-release with or without networks disassembly. Incorporation of bioactive factors into a matrix often goes hand in hand with a slow drug release by migration and the matrix used as scaffold at the same time on a mid-to long-time period. Biobased drug delivery systems have been elaborated with the aim of quick PU degradation for enhanced drug release. It has been achieved by the introduction of PLA [267] or PLGA [268] macromers into the PU backbone. Tailored degradation rates can be obtained by varying the PLA amount or molar mass in the final PU [269]. Another approach is based on PU-grafting onto biopolymers such as chitosan, to develop injectable hydrogel with controlled drug release like known antibacterial drug tetracycline hydrochloride [270,271]. Drug encapsulation in NPs has also been elaborated from sunflower oil diol, IPDI and poly (butylene adipate) diol (PBA diol) using DBTL as catalyst [272]. Raloxifene hydrochloride, an estrogen receptor modulator used for the reduction of breast cancer risks in postmenopausal women, was loaded into PU-based NPs. Controlled in vitro drug release was triggered by hydrolysis and was more stable than raloxifene hydrochloride crude drug solution. Over 350 h sustained release was observed for NPs compared to less than 50 h for the crude solution. Moreover, drug release via NPs depicted excellent antiproliferation properties of cancer cells. Designed solution was hence concluded a promising candidate for the replacement of non-specific intravenous drug injections. Multiresponsive materials, such as redox-, pH-, temperature- or enzymatic-sensitive micelles have also recently been described. The overall concept of bi-responsive drug delivery is described in Fig. 11 with model temperature and enzymatic responsivity. The first stimulus (for example temperature) usually allows drug-loaded micelles insertion in cancer cells, and the second stimulus at intracellular level (in this case enzymatic attack) triggers micelles disassembly and final drug-release. In this regard, biobased self-assembly micelles were prepared from previously synthesized PLA-dithiodiethanol-PLA diol for redox-responsivity [273]. Obtained spherical micelles were stable at neutral pH (7.4). Drug-release was increased with weak acidic pH usually found for instance in cancer cells microenvironment. Other degradation stimuli have been explored and focused on enzymatic intracellular responsive biobased NIPU micelles for anticancer drug release [274]. Such a work opened new horizons for the elaboration of amino acid-based drug release nanocarriers. The same group went further by modifying l-Tyrosine monomer via phenolic O-PEG substitution [275] to develop dual thermo- and enzymatic response of the new micelles, induced by outer-shell PEG thermal phase transition from hydrated to non-hydrated state above its lower critical solution temperature (LCST). The LCST was close to cancer cells microenvironment temperature. Thus, a disassembly of the micelles was observed at this temperature. Then, esterase enzyme was intracellularly acting to release the drug into the cancer cells. The same dual-responsive method was recently developed with a close approach [276].

#### 5.4.2 Tissue adhesives

Tissue adhesives have gain attention in the last decades mainly due to the increasing amount of surgical procedures in the world. Nowadays golden procedure for binding tissues together still consists of suturing or stapling, for instance to avoid tissue bleeding but also to physically attach tissues together for further healing. However, these invasive techniques can lead to complications such as further uncontrolled bleeding or air/gas leaking. In response to that, the emergence of new types of tissue closure have been developed either to replace sutures or to be used in addition with them. These tissue adhesives are divided into three main categories [277] depending on the adhesive purpose: (i) Hemostats, that stop bleeding by intervention in the coagulation cascade [278], usually they offer poor mechanical properties and are usually used in case of blood loss due to sutures tissue damages, (ii) Sealants are mostly used in the case of air/gas or blood leaking by creating a physical barrier [277]. They usually offer mid-range adhesion to tissues and are used in complement of sutures, and (iii) glues, that strongly adhere to tissues [279]. However, these systems work better in a dry environment.

The main requirements for tissue adhesives are adhesion to the final tissue in a wet environment, tissue-adapted mechanical properties, fast in situ curing time (within minutes), limited or no swelling, and biocompatibility while applying and during degradation. Up until now, only one PU-based tissue adhesive is commercially available, TissuGlu® by B. Braun. It is one of the tissue adhesive world market leader. The surgical adhesive is a lysine-derived PU exhibiting strong tissue adhesion and indicated for abdominoplasty interventions [280].

Polymeric adhesives have been extensively studied and reviewed in the last decade [277,281], PUs being only one family of the explored polymers. The key for PU-based tissue adhesive is the –NCO reactivity against water and hydroxyl groups to promote tunable tissue adhesion in wet environment. Biobased –NCO terminated PU adhesives [282,283] have consequently been developed and can be cured in the presence of water and/or a chain extender, thus be used for several types of tissues. Of course, the adhesion strength strongly depend on the tissue substrate [284]. However, PU adhesives major drawback is the relative long curing time [285,286]. To overcome this issue, avoid potential cytotoxicity and the exothermic reaction between –NCO functions and water, adhesives from saccharide-based PU solutions in absolute ethanol have been developed [287]. In this work, no free –NCO functions are present in the material and adhesion was obtained by hydrogen bonding thanks to the numerous –OH groups from saccharide. Another way to improve the curing time is by preparing photo-crosslinkable networks composed of biobased precursors such as methacrylate end-capped PLA [288] or oxidized urethane-modified dextran [289]. In these cases, curing was obtained within minutes. However, the systems described in the literature, as well as commercially available tissue adhesives do not bring the ideal answer to all the basic requirements, hence a lot of research still needs to be performed for PU adhesives to be used as surgical adhesive. Only a few systems are based on renewable biobased PU solutions compared to the large variety of fossil-based PU tissue adhesives recently developed [[290], [291], [292]].

### 5.5 Last innovations on the field of biobased PU for biomedical applications

**Fig. 12:**
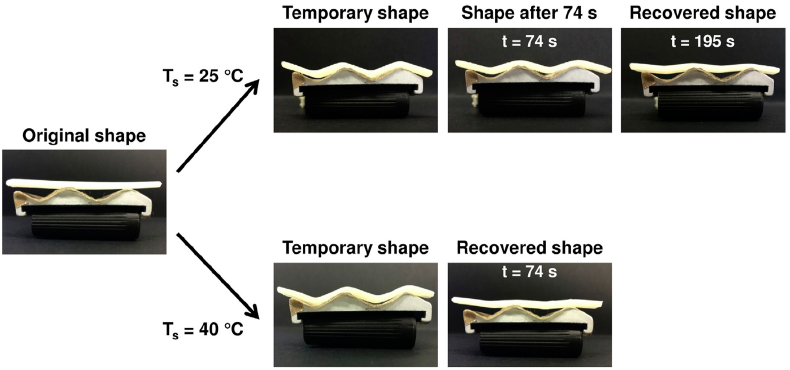
Photographs of SMPU first shape-recovery cycle over time at two different switching temperatures (Ts = 25 °C and 40 °C). From [297].

Last trends in biobased PUs for biomed applications are mainly focused on advanced materials such as stimuli-responsive materials like for example shape-memory structures. Main shape-memory materials for biomedical applications remain in the orthopedic and orthodontic field as wires, or more generally in the surgical area as stent, staple, coils or valves [293]. SMPUs have recently gained a lot of interest as alloys replacements for the same applications and in tissue engineering due to their adaptable transition temperatures via the soft-hard domain tunability, as well as lower density and cost, and higher deformation strain than shape-memory alloys [294]. As previously mentioned, these stimuli-responsive smart materials can recover their primary shape after being fixed in a second shape, which promotes minimally invasive surgical procedures. The recovery to the primary shape can be triggered by different stimuli such as water [295], via a magnetic field [296] or in most cases by temperature [297,298] and are called switch temperatures (Ts). For thermally responsive SMPU, the transition is usually triggered by the crystalline melting point of the SS, or the Tg for amorphous PUs. Shape-recovery photographs at different times of a temperature-responsive SMPU at two different Ts are depicted in Fig. 12. To be used as implant, SMPU should meet certain requirements to avoid tissue damage or premature shape recovery: they should have a high and quick shape recovery near body temperature, as well as be biodegradable and biocompatible. Thus, the research has been focused on designing PUs with such properties. Biobased PLA [299,300]- and vegetable oil [297,301,302]-based polyols are pertinent choices to synthesize renewable SMPUs. Moreover, polysaccharides have also been studied as multi-branched polyols [303], as well as biobased polycarbonate diol [304] and polyester polyols like PGS diol [295], PEA and PBA diols [305]. Important SMPUs properties of the previously mentioned studies and regarding their potential uses in the biomedical area reported in Table 6. Studies revealed consequent shape recovery. However, only few studies investigated the in vitro bio- or cytocompatibility of their materials, and even less studied their biotic degradation, despite these points being key parameters for biomedical purposes. In the above-mentioned works, only two of them evaluated the biotic degradation of their SMPUs [295,303]: the multibranched SMPU films degradation in PBS at 37 °C with *P. aeruginosa* bacterial for 6 weeks has been studied. They found weight losses of 5–30 wt%, which would allow the SMPU to be used for long term applications. Biotic degradation studies was also studied [295] in enzyme-free and lipase-induced PBS at 37 °C and pH 7.4, for 28 days which resulted in less than 10 wt% for the enzyme-free degradation, and up to 27 wt% in lipase-induced environment.

**Table 6:** Main biobased SMPUs and recovery properties.

Ref	Polyol type	Thermal transition	Shape memory parameters		Shape memory (cycle 1)*		Recovery test		Cytotoxicity
			Tdef, Tfix, Trec**	Streching [%]	Shape Fixity Rf [%]	Shape Recovery Rr [%]	Temp.***	Time	Test type, Cell type
[302]	CO	Tm: 20.5 °C–54.4 °C	65 °C, 5 °C, 65 °C	n.m	47.8–94.6	82.0–94.1	n.m	n.m	MTT, NIH 3T3
			65 °C, 25 °C, 65 °C		57.4–97.7	88.6–96.7	n.m	n.m	
[297]	CO	Tm: 65.4 °C–70.0 °C	n.m, n.m, 25 °C	50	78.1–93.5	90.5–95.6	25 °C	195s	MTT, L929
			n.m, n.m, 40 °C		87.1–95.5	91.1–94.5	40 °C	74s	
[301]	CO	Tm: 52.2 °C–65.0 °C	>Tm, <Tm, >Tm	50	88.9–96.9	81.7–93.1	n.m	n.m	MTT, L929
				100	86.4–88.6	78.1–85.0	n.m	n.m	
				250	72.0–80.0	72.4–84.7	n.m	n.m	
[296]	CO	Tg: 13.6 °C–14.5 °C	40 °C, −10 °C, 40 °C	50	91.6–98.3	85.5–87.3	n.m	n.m	Optical density, L929
[299]	PLA-PCL-PLA	Tg: 38.9 °C–46.8 °C	60 °C, 0 °C, 60 °C	n.m	99.4–99.9	close to 100	60 °C	<10s	n.m
[298]	PLA-co-CL	Tg: 28.7 °C–34.0 °C	37 °C, −195,79 °C, 37 °C	200	n.m	43–52	37 °C	1–2min	n.m
[300]	PLA	Tg: 41.1 °C–54.1 °C	Tg+15 °C, 40 °C, 75 °C	100	>95	>70	60 °C	10s	n.m
[303]	Starch	Tm: around 40 °C	60 °C, −15 °C, 40 °C	n.m	70.0–98.8	93.3–98.9	40 °C	20–25s	Live/Dead, HDF
[304]	PC	Tm: 37 °C–54 °C	60–70 °C, -10-0 °C, 60–70 °C	100	93.3–98.1	98.6–99.3	n.m	n.m	n.m
				400	91.8–98.4	98.5–99.3			
				600	90.9–97.7	99.1–99.4			
[305]	PBA	Tm: 44.6 °C–47.3 °C	40 °C, 10 °C, 40 °C	50	75–85	75–80	60 °C	50s	n.m
		Tm: 43.6 °C–48.6 °C			70–75	70–75			
	PEA	Tm: 41.3 °C–42.3 °C			70–80	70–75			
[295]	PGS	–	in water, 24 °C	–	25–90	70–95	22 °C	<1 h	n.m
			in PBS, 37 °C		25–90	75–90			

N.m: not mentioned *Most works comprised several cycles **Tdef = deformation temperature, Tfix = fixation temperature, Trec = recovery temperature ***Temp. = temperature.

## 6 Summary, perspectives and future trends

This review has provided an overview of the trends on biobased PU for biomedical applications. During the last two decades, biobased PU have achieved significant progress for general and specific biomedical applications such as, stimuli responsive SMPUs, which have demonstrated great potential for metal alloys replacement with improved properties.

For implantable biomaterials, PUs were carefully designed to undergo several degradation types over various periods in contact with living tissues. However, the more advanced and numerous requirements, the more unique its structure becomes, which makes it almost impossible to put forward general statements regarding the degradation products toxicity. Degradation rate and the tissue ability to avoid small molecules accumulation by moving them to metabolizing organs are key parameters that need in vivo assessment to be fully accepted as biocompatible. In this frame, while many studies demonstrated a general PU cytocompatibility, biocompatibility at usage and degradability, there still lack a clear characterization of the degradation products and their corresponding toxicity levels. These analyses are particularly expensive and time-consuming, then, they are reserved mainly for advanced projects. Moreover, regarding other potential toxicity origins, the use of organometallic catalysts with proven toxicity are still golden standards for PU syntheses. While in catalytic quantities, they can still affect the overall PU biocompatibility either at usage or while degrading, and their replacement should be considered when possible.

One should emphasize that renewable alternatives to fossil-based PU biomaterials are still an emerging technology, from which preliminary results were here described. Starting from the studies results analyses, we can show that they exhibited great potentials for stimulating future developments. However, main lacks of these studies are sometimes i) a too low biobased content, ii) the specific formulations effects on human tissues understanding and iii) the knowledge of residual catalyst toxicity and of the degradation products.

Finally, the future trends in biobased PUs for biomedical applications lie in the development of fully biobased materials with biomimetic properties. For that, smart structures like for instance SMPUs, or CANs such as vitrimers with tunable dynamic covalent bonds could be of strong interest in the coming years. These structures indeed need to be investigated for biomedical purposes like, for instance, for wound dressings. They could offer advanced and adaptable properties in terms of mechanical resistance, degradability and recyclability compared to current systems.

## Declaration of competing interest

The authors declare no conflict of interest.
